# A systematic review on the impact of delayed local therapy in patients with Ewing sarcoma of the pelvis

**DOI:** 10.1007/s00432-025-06286-8

**Published:** 2025-08-27

**Authors:** Shook Fe Yap, Natacha Omer, Vivek Bhadri, Jeremy Lewin, Wayne Nicholls, Claire Carkeet, Lisa Orme, Marianne Phillips, Mark Winstanley, Smaro Lazarakis, Jasmine Mar, Angela Hong, Julie Cayrol

**Affiliations:** 1 Oncology Services Group, Queensland Children’s Hospital, South Brisbane, QLD 4101 Australia; 2 Faculty of Medicine, The University of Queensland Frazer Institute, The University of Queensland, Woolloongabba, QLD 4102 Australia; 3 Central Clinical School, Faculty of Medicine and Health, The University of Sydney, Sydney, NSW 2060 Australia; 4 Department of Medical Oncology, Chris O’Brien Lifehouse, Camperdown, NSW 2050 Australia; 5 Department of Cancer Medicine, Peter MacCallum Cancer Centre, Parkville, VIC 3010 Australia; 6 Victorian Adolescent & Young Adult Cancer Service, Parkville, VIC 3010 Australia; 7 Sir Peter MacCallum Department of Oncology, The University of Melbourne, Parkville, VIC 3010 Australia; 8 Children’s Cancer Centre, The Royal Children’s’ Hospital Melbourne, Parkville, VIC 3052 Australia; 9 Department Oncology, Haematology and Tissue & Cellular Therapy, Perth Children’s Hospital, Perth, WA 6009 Australia; 10 The Kids Research Institute, Nedlands, Perth 6009 Australia; 11 Starship Paediatric Blood & Cancer Centre, Grafton, 1142 Auckland New Zealand; 12 Royal Melbourne Hospital, Parkville, VIC 3010 Australia; 13 Australia and New Zealand Sarcoma Association, Melbourne, VIC Australia; 14 Department of Radiation Oncology, Chris O’Brien Lifehouse, Missenden Road, Camperdown, NSW 2050 Australia; 15 The Royal Children’s Hospital Melbourne, Parkville, VIC 3010 Australia; 16 Murdoch Children’s Research Institute, Parkville, VIC 3010 Australia

**Keywords:** Ewing sarcoma, Sarcoma, Surgery, Radiation therapy, Radiotherapy, Guidelines

## Abstract

**Background:**

Local treatment of pelvic Ewing Sarcoma (EWS) is czhallenging due to complex anatomy and potential complications. Local therapy may be deferred to maintain chemotherapy dose-intensity, but the impact of this delay on outcomes remains unclear.

**Methods:**

A systematic review using the PICO model evaluated whether delayed local therapy affects long-term outcomes in localized pelvic EWS. Ovid Medline, Ovid Embase, and Cochrane Central databases were searched through June 2024. Studies were screened by two independent reviewers for quantitative and qualitative data extraction.

**Results:**

Of 1849 studies screened, eight eligible studies including 2618 patients were identified; none were pelvic-specific. When specified, 1–18% had pelvic primaries. All studies were retrospective with substantial heterogeneity in endpoints. Seven of eight studies indicated delayed local therapy was associated with reduced event-free survival (EFS) and/or overall survival (OS). In the largest study (Lin et al.) of 1319 EWS patients, delayed local therapy > 16 weeks was associated with significantly reduced OS (HR 1.41, CI 1.11–1.80, *p* = 0.005).

**Conclusion:**

This systematic review highlights the importance of multidisciplinary coordinated care during initial chemotherapy to optimize local therapy timing, which may impact outcomes. Further studies are needed to assess timing significance, particularly for pelvic EWS.

**Supplementary Information:**

The online version contains supplementary material available at 10.1007/s00432-025-06286-8.

## Introduction

Ewing sarcoma (EWS) is a rare and aggressive malignancy characterized by small round blue cells arising from bone and soft tissue. It is the second most prevalent bone tumour in children, adolescents and young adults [1–3]. With the current chemotherapy dose-intensive 2-weekly regimen, the 5-year overall survival (OS) and event-free survival (EFS) rates for patients with localised EWS are 87% and 78%, respectively [4]. These positive outcomes can be attributed to the progressive use of multimodal therapy and advances in surgical and radiotherapy techniques [5]. The overall clinical outcome for metastatic or recurrent EWS remains generally poor [6, 7]. However, patients with lung metastases only have an OS of 68% compared to those with multi-metastatic disease with an OS of 28% [6, 8].

Treatment of EWS involves the administration of intensive multi-agent chemotherapy, followed by local therapy and further consolidation chemotherapy. Local therapy may include surgery, radiotherapy, or a combination of both. In the Children’s Oncology Group (COG) and Euro-Ewing protocols, the timing of local therapy is recommended after 6 cycles (12 weeks)[4] or 9 cycles (18 weeks)[9] of chemotherapy respectively, with careful planning amongst a sarcoma multidisciplinary team [4, 6, 9]. Radiotherapy may be administered in the preoperative setting for patients at high risk of incomplete resection due to tumour location or size, or in the postoperative setting for patients with positive surgical margins or unfavourable histological responses to chemotherapy. The optimal approach for local control is influenced by variety of factors, including patient’s age, tumour location, tumour size, and local extension[3, 10–12].

While EWS commonly arises in the long bones, approximately 26% of EWS cases arise in the pelvis, which presents unique challenges in diagnosis and treatment [3]. Pelvic EWS has been associated with less favourable outcomes compared to other skeletal sites [5, 7, 13–15]. As reported by Ahmed et al., the 5-year OS and EFS for patients with localized pelvic EWS were 73% and 65% respectively, while those rates were significantly lower, 30% and 18%, respectively, for patients with metastatic pelvic EWS [14]. Most studies report that patients with pelvic EWS tend to experience earlier relapses, with higher rates of local recurrence, and lower disease-free and OS rates [12, 13, 15–17]. These patients are more likely to present with larger tumour volumes and are more likely to have metastatic disease at diagnosis [8, 12, 15, 18]. Complex resections in this location, and limited reconstructive options lead to higher risks of complication, such as delayed wound healing and secondary infection [2, 5], which can impact the provision of ongoing post-operative chemotherapy and create significant delays in dose-intensive treatment. Furthermore, deferring surgical resection of the tumour until the end of treatment can allow more time for surgical planning, the printing of custom 3-D implants and potential further tumour volume reduction. In this context, some oncologists and orthopaedic surgeons may elect for certain patients to complete chemotherapy before surgery.

Over the past decade, several publications have compared treatment modality for local control (radiotherapy vs. surgery alone, or combination) in patients with pelvic EWS; however, the optimal timing of local therapy after initiation of chemotherapy in this setting remains unclear [13, 15, 17–19]. Given the limited data available in the literature, the Australia and New Zealand Sarcoma Association (ANZSA, www.sarcoma.org.au) Clinical Practice Guidelines Working Party conducted a systematic review, aiming to provide an evidence-based set of recommendations and practice points on the optimal timing of local therapy for patients with pelvic EWS.

## Methods

Clinical practice guidelines for sarcoma were developed by a multidisciplinary working party established by ANZSA. Key clinical questions relating to paediatric, adolescent and young adult patients with sarcoma were identified. One of the questions was to evaluate the impact of delayed local therapy in patients with localised pelvic EWS. A systematic review using the following PICO (patient/population, intervention, comparison and outcome) model was conducted to respond to this question. The detailed protocol for this review was registered and published on the PROSPERO register (registration number CRD42022277484).

*P*opulation: Patients with localised pelvic EWS of any age.

*I*ntervention: Delayed local therapy of the primary tumour compared to recommended time-point as per protocol.

*C*omparison: Local treatment at recommended time-point as per protocol.

*O*utcomes: Local recurrence rate, overall survival, event-free survival, surgical complications, length of peri-operative admission, limbs salvage rate, functional outcome.

A systematic search for evidence was undertaken in June 2023, and updated in June 2024, in the following electronic databases: Ovid Medline, Ovid Embase, Cochrane CENTRAL (Wiley) using the search strategy outlined in Supplementary Fig. 1. The search strategy was developed after having identified Medical Subject Headings (MeSH) terms and keywords from key publications on this topic. It was refined and approved by the members of the Working Party, adapted to the different databases and pilot-tested by ensuring sentinel publications could be identified with the search. Date of coverage was restricted to 1990 onwards and searches were limited to articles in English. Randomised and non-randomised controlled studies were included if they reported the impact on outcome of timing of local therapy (surgery alone, radiotherapy alone or surgery with radiotherapy) from initiation of chemotherapy in patients with localised pelvic EWS. Case reports, conference abstracts and review articles were excluded. The literature review management software Covidence was used to facilitate the conduct of the review [20]. Each study was screened by title and abstract for eligibility as per the PICO model, and against the inclusion and exclusion criteria by two independent reviewers (AH, CC, NO, SFY). The full text of each study was then assessed for eligibility by two independent reviewers (AH, JC, NO, SFY). A reason for exclusion was assigned to each excluded study. Any conflict between two reviewers was resolved by the lead of the clinical question (NO) and through discussion.

Quantitative data extraction for each study was performed in Covidence by a member of the guidelines working party using a custom-made data extraction template (Supplementary Fig. 2) and verified by a second reviewer. The quality of each study was assessed by two independent reviewers using the NHMRC Evidence Hierarchy [21] and Newcastle-Ottawa Quality Assessment Form for Cohort Studies [22]. A final score for the quality assessment was assigned to each study. An evidence table which summarised the systematic assessment and critical appraisal of all studies meeting the inclusion criteria was created for the working party to appraise, along with an evidence statement form which documented the synthesis and evaluation of the body of evidence to determine the grade of the recommendation according to an NHMRC-approved method [21].

## Results

**Fig. 1:**
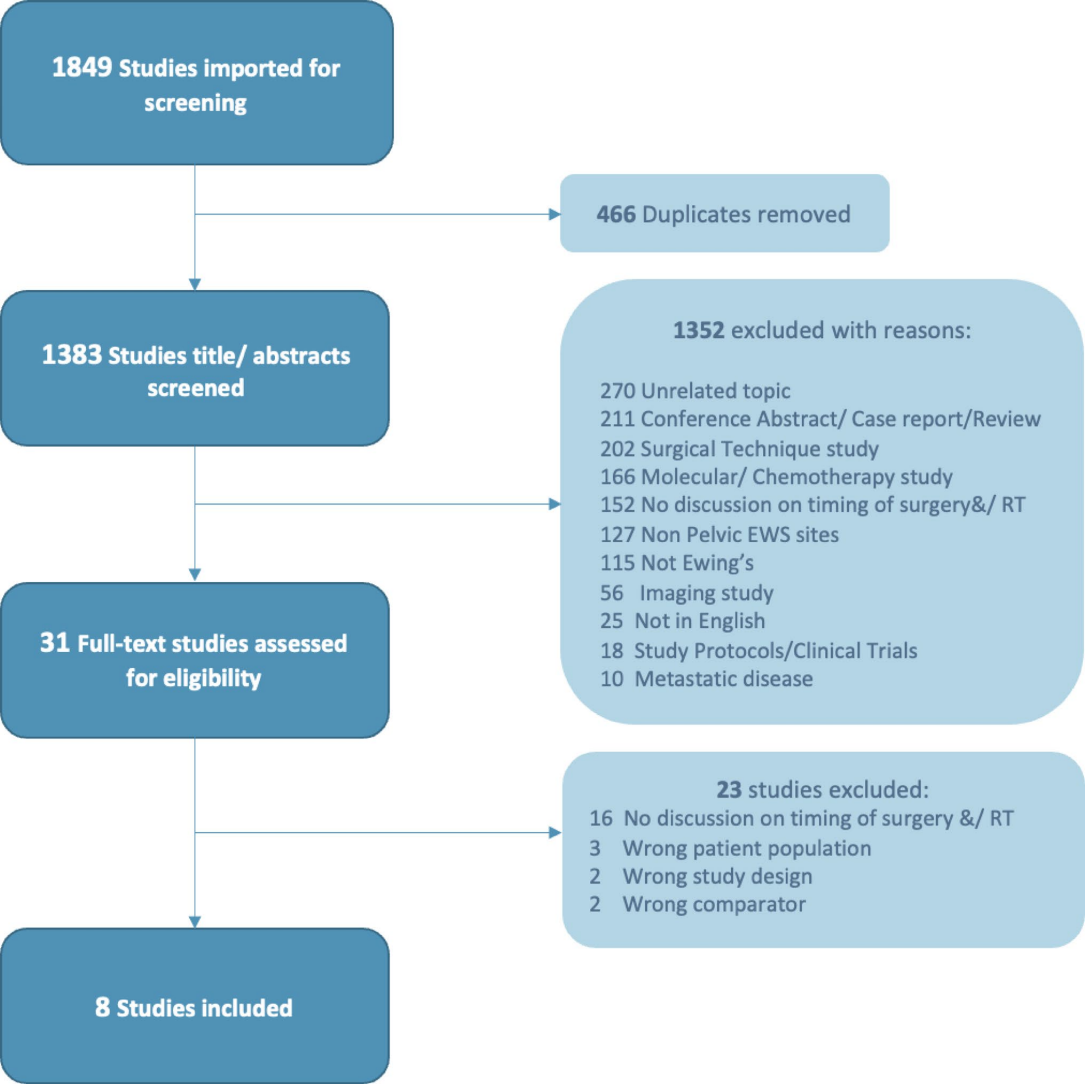
PRISMA flow diagram of the systematic review

The PRISMA flow chart shows the different screening phases of the systematic review (Fig. 1). A total of 1849 studies were identified from the search strategy and imported into Covidence for screening. After removing duplicates, a remaining 1383 studies were screened according to the PICO model. The selection process resulted in a eight studies for systematic review [23–30]. The quality of each study and an evidence statement form can be found in supplementary Tables 1 and 2. All eight studies were NHMRC Evidence hierarchy Level III-3, with five studies rated good quality on the Newcastle-Ottawa Quality Assessment scale.

All eight studies were retrospective cohort studies with substantial heterogeneity in primary endpoints (Table 1). No study was specific to pelvic EWS and only five studies detailed the number of patients with pelvic EWS in their publications. Five studies included timing of local therapy as a variable in multivariable analysis.

**Table 1:**
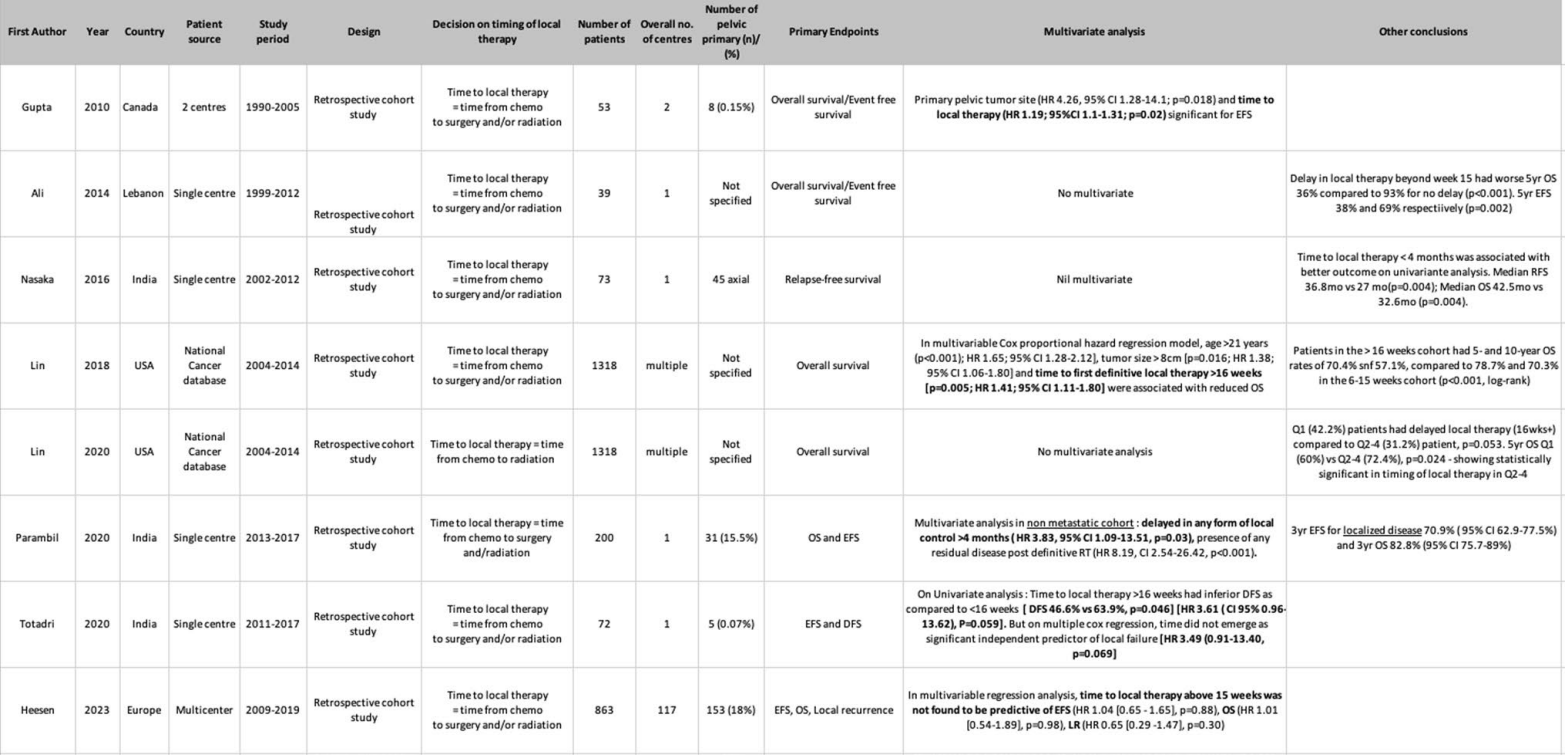
Characteristics of the eight studies included

OS, overall survival; EFS, Event-free survival; HR, hazard ratio; CI, confidence interval; y, years; mo, months; w, weeks; PD, progressive disease; RFS, relapse-free survival

## Delayed local therapy with surgery and/or RT

A delay in local therapy beyond 16 weeks or 4 months from initiation of chemotherapy was associated with reduced EFS and/or OS in seven of the eight studies included. The largest study was from the United States National Cancer Database including 1318 patients with localised EWS of unspecified location [27]. The overall survival was significantly improved in patients who commenced local therapy (surgery and/or RT) 6 to 15 weeks after initiation of chemotherapy compared to those who started after 16 weeks (78.7% vs. 70.4%, and 70.3% vs. 57.1% at 5 and 10 years respectively, *p* < 0.001). This difference in survival when local therapy was delayed was the largest in patients receiving radiotherapy alone (5-year OS 80% vs. 58.9%, *p* = 0.03). In the multivariable Cox proportional hazards regression model, time to first definitive local therapy beyond 16 weeks was associated with reduced overall survival with a HR of 1.41 (95% CI 1.11–1.8, *p* = 0.005). Age > 21 years (HR 1.65, 95% CI, 1.28–2.12, *P* < 0.001), tumour size > 8 cm (HR, 1.38, 95% CI 1.06–1.80, *P* = 0.016) were also associated with reduced overall survival [27]. However, details of the chemotherapy regimen used in this cohort were not mentioned in the study.

Parambil et al. analysed 31 out of 200 patients with primary pelvic EWS in their study. This retrospective study included patients under 15 years of age, treated in a single centre in India using an institutional Human Ethics Committee-approved, non-dose intense chemotherapy protocol (EFT-2001). This protocol consisted of alternating combination drugs (Vincristine-Ifosfamide-Etoposide (VIE) and Vincristine-Actinomycin-Cyclophosphamide (VAC)) administered on a 3-weekly schedule, except for an interval compression in induction between two Vincristine-Doxorubicin-Cyclophosphamide (VDC) cycles. Local therapy was scheduled after 9–12 weeks of chemotherapy and local therapy modality (surgery and/or radiotherapy) decided by a multidisciplinary team. The total duration of the regimen was 42 weeks if local therapy involved surgery and 46 weeks if definitive radiotherapy was administered. In multivariate analysis, in the non-metastatic cohort (with a reported 3-year EFS of 70.9%), delay in any form of local therapy beyond 16 weeks of induction chemotherapy led to an inferior EFS (HR 3.83, 95% CI: 1.09–13.51, *p* = 0.03 in the non-metastatic cohort) [28].

Similarly, a retrospective analysis conducted by Gupta et al. reviewed 53 patients of both adult and paediatric cohorts with localized EWS, including eight pelvic cases, across two centers in Canada: The Hospital for Sick Children (Sickkids) and Mount Sinai Hospital (adult), covering the period from 1990 to 2005. The chemotherapy regimens and dosing strategies employed at both institutions were consistent, featuring a combination of VDC alternating with IE administered every three weeks. Prior to 1996, patients at SickKids received treatment with the intent of completing 10 cycles, with local therapy scheduled to commence after the 4 cycle (12 weeks). From 1996 onward, patients at SickKids were treated in accordance with the Intergroup Study 0154 protocol, which included 5 cycles of VDC and 4 cycles of VC alternating with IE, for a planned total of 17 cycles. Local therapy was planned after 12 weeks of therapy [23]. In this small cohort, multivariate analysis demonstrated that primary pelvic site (HR 4.26, 95% CI 1.28–14.1, *p* = 0.18) and time to local therapy (HR 1.19, 95% CI 1.1–1.31, *p* = 0.02) had an unfavourable impact with a 3-year EFS in both the paediatric and adult patients of 70% ± 9% and 43% ± 13%%, respectively [23].

Three other publications from single centre institutes reported similar outcomes. Ali et al. conducted a study involving 39 patients with EWS of unspecified location in Lebanon. Prior to 2009, all patients in this cohort were uniformly treated following the POG9354/CCG7742 Regimen A. Following 2009, treatment protocols transitioned to COG AEWS0031 Regimen B, which included interval compressed chemotherapy cycles. Definitive local control was attempted at 12–15 weeks of treatment [24]. In their analysis, utilizing a univariate Cox regression model, they found that delayed local therapy beyond 15 weeks after initiation of chemotherapy correlated with decreased 5-year EFS and OS, with hazard ratios of 16.123 (95% CI: 1.996-130.231, *p* = 0.009) and 5.002 (95% CI: 1.653–15.135, *p* = 0.004) respectively [24]. Totadri et al. conducted a retrospective study in India involving 48 newly diagnosed patients with EWS under the age of 15 including 5 pelvic EWS. They employed a modified version of the Intergroup study INT-0091 protocol, which included VDC and IE chemotherapy administered every three weeks over a total duration of 48 weeks, with local therapy planned following radiological reassessment at 12 weeks post-initiation of therapy [29]. In their univariate analysis, they found that time to local therapy beyond 16 weeks associated with inferior disease-free survival (*p* = 0.046), however this was not statistically significant on multiple regression analysis [HR 3.49 (0.91–13.40, *p* = 0.069] [29]. Nasaka et al. analysed 73 patients with localized EWS at a single-centre institution in India comparing various treatment protocols. These included a comparison of three groups: [1] those who received non-ifosfamide-based regimens (VDC/vincristine, actinomycin-D, cyclophosphamide/vincristine, actinomycin-D, cyclophosphamide, and doxorubicin [2], those who received VDC/IE administered over 12 cycles, and [3] those who received VDC/IE for 17 cycles. Local therapy, either surgery or radiotherapy, was applied after a few of neoadjuvant chemotherapy. The study included 45 patients (62%) with an axial skeleton primary, and reported a superior 3-year relapse-free survival (17% vs. 60%) and 3-year OS (35% vs. 70%) in those who had no delay in local therapy of more than 4 months on univariate analysis (*p* = 0.004) [25]. The authors also observed that patients receiving VDC/IE for 17 cycles coupled with shorter time to local control demonstrated better outcomes than the other groups. All of these three studies were conducted with a limited sample size (*n* = 39–73) and were unable to control for factors that may influence treatment, prognosis, and disease control in pelvic EWS.

In contrast to the previously mentioned studies, the research conducted by Heesen et al. [30] presents different findings. This study investigated the relationship between local therapy and event-free survival, overall survival, and local recurrence in patients with localized EWS, utilizing data from the international EWING 2008 study conducted across 117 centers in 13 countries. The study included a total of 863 patients, of which 153 (17.7%) had localized pelvic EWS, while the majority (41.4%) had extremity EWS [30]. Patients received 6 cycles of induction chemotherapy consisting of vincristine, ifosfamide, doxorubicin, and etoposide (VIDE) given 3-weekly, followed by local treatment and consolidation chemotherapy. Consolidation therapy included 8 cycles of either vincristine, actinomycin D, and ifosfamide (VAI) or vincristine, actinomycin D, and cyclophosphamide (VAC), or one course of VAI and high-dose chemotherapy with busulfan and melphalan. Although the recommended length of induction chemotherapy was 18 weeks in this trial (six 3-weekly cycles), the authors analysed the impact of timing of local therapy before or after 15 weeks. Analysis of the entire cohort revealed no statistically significant difference in 5-year EFS between patients who received local therapy after 15 weeks [0.7 (0.67,0.74]) compared to within or less than 15 weeks (0.74 [0.65, 0.84]), *p* = 0.70 [30]. Similarly, no significant differences were observed in 5-year OS (*p* = 0.71) or 5-year local recurrence rates (*p* = 0.31) [30]. Furthermore, the multivariable regression analysis indicated that delay in local therapy of more than 15 weeks was not predictive of EFS [HR 1.40 (0.65–1.65), *p* = 0.88], OS [HR 1.01 (0.54–1.89), *p* = 0.98], or local recurrence [HR 0.65 (0.29–1.47), *p* = 0.30] [30].

## Delayed local therapy with radiotherapy only

Lin et al. examined the relationship between treatment centre volume and the timing of definitive radiotherapy. The findings indicated that delayed local therapy with definitive radiotherapy occurring more than 16 weeks after the initiation of chemotherapy, was associated with statistically significant reduced overall survival in lower volume centres (Q1) compared to higher volume centres (Q2-4) (*p* = 0.024) [26]. However, among the higher quartile cohorts, the difference in the delay of initiation of RT for Q4 vs. Q2-3 was not statistically significant (*p* = 0.153) [26]. These findings should be interpreted with caution, as the improved survival rates observed at high-volume treatment centres may result from multiple factors, including variations in chemotherapy regimens and supportive cares, level of sarcoma expertise within the multidisciplinary team, and the coordination of care, which were not captured for analysis in the study [26]. The authors also acknowledged confounding factors such as disease burden, insurance status, and income, which may have influenced the choice of treatment centre [26]. 

Finally, the other outcome endpoints in the PICO model of the systematic review (local recurrence rate, surgical complications, length of peri-operative admission, limb salvage rate, functional outcome) were not consistently reported in these studies for appraisal.

## Discussion

This systematic review aimed to determine the impact of timing of local therapy in patients diagnosed with pelvic EWS. Of 1849 studies screened, the search identified eight relevant studies, including 2618 patients, addressing this question. All eight studies included all primary locations, and when specified, the number of patients with pelvic primary was usually small (1–18% of included patients). Albeit with limited and heterogeneous data and no specific analysis of the location of interest, seven of the eight relevant studies included were consistent in identifying inferior survival when local therapy was delayed beyond 16 weeks from the initiation of chemotherapy. In the multivariable analysis of the largest study, time to first definitive local therapy beyond 16 weeks was associated with reduced overall survival with HR of 1.41(*p* = 0.005) [27]. Likewise, Parambil et al. examined the second largest pelvic cohort within the paediatric population and demonstrated delayed local therapy beyond four months correlated with an inferior EFS with a HR of 1.73 (*p* = 0.03) [28]. The delay in local therapy could be attributed to several factors, including the need for advanced surgical planning due to the complex anatomy, the availability of dedicated sarcoma surgeons or radiation oncologists or facilities needed for local therapy, poor tolerance to induction chemotherapy, the hope that additional chemotherapy might achieve greater tumour reduction, or patient preference related to cost or potential morbidity associated with surgery. There may be an interaction between these factors and survival that this systematic review is unable to determine.

Heesen et al. was the only study to have demonstrated, based on the Euro-Ewing 2008 trial, that the timing of local therapy did not affect EFS (HR 1.04), OS (HR 1.01), or local recurrence (HR 0.65) in their multivariable analysis [30]. However, the authors compared patients who underwent local therapy < 15 weeks after the start of chemotherapy to after 15 weeks. We would expect the first group to be very small considering induction therapy consisted of six 3-weekly cycles of chemotherapy. As such, patients would not be expected to be ready for surgery before weeks 17–18, and the majority of patients with early local control might be those receiving radiation therapy only.

The existing literature offers limited information regarding the impact on survival of the timing of local therapy for EWS, and our systematic review is the first studying this question. As discussed above, most of the identified studies demonstrated that a delay in local therapy impacts EWS outcome. Even though our search yielded only eight retrospective publications including patients with the tumour site of interest, this was considered acceptable by the working party considering the rare nature of EWS, particularly in the specified location, and the focus on local therapy modality rather than timing in recent studies. Interestingly, another publication by Samer et al. including 43 patients with localised extremity EWS also demonstrated an impact of timing of local therapy on survival outcome. OS was significantly lower in patients with delayed local therapy beyond 15 weeks, with a 5-year OS rate of 56% compared to 80% (*p* = 0.044) [31].

Nevertheless, our review has several limitations. All eight studies encompassed various primary sites and the number of patients with pelvic primaries was limited. Interpretation of the results may be complicated by confounding factors associated with these various tumour sites, variable local therapy modalities and reasons for the different timing of the local therapy. Further, the specific chemotherapy protocols used can also influence the final outcomes. The introduction of interval compressed chemotherapy, as demonstrated in the AEWS0031 trial [32], has led to improved outcomes for patients with localized EWS compared to historical trials. In the large study by Lin et al., the authors did not disclose specific details regarding the treatment regimen utilized for this cohort. This prompts a discussion on the potential influence of different chemotherapy regimens on the reported improvements in overall survival observed. Finally, the heterogeneity of the studies, substantial difference in number of patients included, and the absence of common reported clinical outcome also prevented us from being able to aggregate their results in a meta-analysis to support a definitive conclusion.

The AEWS1031 trial conducted by the Children Oncology Group (COG), utilised a regimen of 17 cycles of interval-compressed chemotherapy with five different drugs randomising VDC vs. VTC chemotherapy. It demonstrated comparable 5-year EFS rates for patients with pelvic primaries and non-pelvic primaries, at 75% and 78% respectively [4]. This trial incorporated local treatment at week 13 of the protocol, which may have contributed to the improved outcome seen in patients with pelvic primaries. The EURO EWING 2012 (EE2012) protocol compared two different strategies for induction and consolidation therapy. Specifically, local control was provided after either 6 cycles of VIDE (Group 1) or 9 cycles of VDC/IE (Group 2), totalling 18 weeks of treatment. The results from the EE2012 protocol indicated inferior EFS and OS rates with the VIDE regimen (3-year EFS of 61% vs. 67%, adjusted HR 0.71 (0.55–0.92)), along with a higher incidence of chemotherapy-related toxicity, underlining the influence of the chemotherapy regimen on outcomes [6]. While the outcome data using a similar induction chemotherapy regimen (VDC/IE) but a different local control timepoint in the EE2012 study was slightly worse compared to the AEWS0031 COG study (5-year EFS 73% in VDC/IE group) [32], it is important to note that the EE2012 cohort included patients with metastatic disease, making this difference in outcome difficult to attribute to the different timing in local therapy (12 vs. 18 weeks) as a causative factor. Now that interval compressed chemotherapy with VDC/IE is internationally recognized as standard of care for EWS, this could allow to better study the impact of timing of local therapy on outcome in different primary locations.

## Conclusion

The existing evidence indicates that delayed local therapy of EWS beyond 4 months of initiation of chemotherapy may be associated with inferior outcomes. Given the complexity of EWS treatment planning, the ANZSA Guidelines Working Party recommends that the sarcoma multidisciplinary team works closely during the initial chemotherapy phase to coordinate a timely evaluation of disease response and local therapy of pelvic EWS. Where possible, local therapy should be planned within 4 months from the start of chemotherapy or, if patients are enrolled in clinical trials, at the recommended timepoint in the protocol (Table 2). Further prospective studies on EWS are essential to establish more robust evidence concerning the optimal timing for local treatment.

**Table 2:** Recommendations from the Australia and New Zealand sarcoma association

Evidence summary	Level
Delays in local therapy for localised pelvic Ewing sarcoma beyond 4 months from the start of chemotherapy may be associated with reduced overall survival.	III-3

Evidence-based recommendation	Grade
Delays in local therapy (surgery and/or radiation therapy) for localised pelvic Ewing sarcoma beyond the recommended timepoint by treatment protocol should be avoided.	C

Practice point	
Patients with pelvic Ewing sarcoma should be managed within a multidisciplinary team who should work closely during the initial chemotherapy period to coordinate the optimal time for disease response evaluation, restaging imaging, and local therapy as per treatment protocol.	

## Supplementary Information

Below is the link to the electronic supplementary material.

Supplementary file1 (DOCX 20 kb)

## Data Availability

No datasets were generated or analysed during the current study.
