# Supplementary material for: A systematic review on the impact of delayed local therapy in patients with Ewing sarcoma of the pelvis
Source: J Cancer Res Clin Oncol. 2025 Aug 27;151(8):237. doi: 10.1007/s00432-025-06286-8 (PMC12390909; doi:10.1007/s00432-025-06286-8)
Supplement: Supplementary file 1 — Supplementary file1 (DOCX 20 kb) [file 432_2025_6286_MOESM1_ESM.docx]

Supplementary Figure 1. Database search strategy

Database: Ovid MEDLINEI ALL <1946 to July 23, 2021>

Search Strategy:

8 exp Sarcoma, Ewing/ (7226)

9 ewing*.mp. (11908)

10 8 or 9 (11908)

14 (exp time factors/ or timing.mp.) and (surgery or surgeries or surgical or surgeon* or resect* or excision).mp. (213872)

15 ((delay* or postpone* or defer* or local control) adj3 (surgery or surgeries or surgical or resect* or excision)).mp. (11765)

16 14 or 15 (222938)

34 10 and 16 (214)

46 (melanoma* or kaposi* or glioma* or carcinoma* or renal cell or brain or leuk?emia* or cell line* or “in vivo” or “in vitro”).ti,ab. (3953955)

47 exp animals/ not exp humans/ (4864720)

48 (animal* or rat or rats or swine or mouse or mice or dog or dogs or canine*).mp. (7362254)

49 (case reports or systematic review or editorial).pt. (2929761)

50 (case report* or systematic review*).ti,ab. (604288)

51 47 or 48 or 49 or 50 (10323155)

52 46 or 47 or 48 or 49 or 50 (12271310)

60 34 not 52 (151)

Supplementary Figure 2. Data extraction table

| Study Identification |  |  |  |  |  |  |  |  |
| --- | --- | --- | --- | --- | --- | --- | --- | --- |
| Sponsorship source |  |  |  |  |  |  |  |  |
| Country |  |  |  |  |  |  |  |  |
| Setting |  |  |  |  |  |  |  |  |
| Comments |  |  |  |  |  |  |  |  |
| Authors name |  |  |  |  |  |  |  |  |
| Institution |  |  |  |  |  |  |  |  |
| Email |  |  |  |  |  |  |  |  |
| Address |  |  |  |  |  |  |  |  |
| Methods |  |  |  |  |  |  |  |  |
| Design |  |  |  |  |  |  |  |  |
| Group |  |  |  |  |  |  |  |  |
| Population |  |  |  |  |  |  |  |  |
| Inclusion criteria |  |  |  |  |  |  |  |  |
| Exclusion criteria |  |  |  |  |  |  |  |  |
| Group differences |  |  |  |  |  |  |  |  |
| Study period |  |  |  |  |  |  |  |  |
| Source of patients |  |  |  |  |  |  |  |  |
| How the decision on timing of local surgery was made? |  |  |  |  |  |  |  |  |
| Follow up |  |  |  |  |  |  |  |  |
| Number of patients |  |  |  |  |  |  |  |  |
| Univariate or multivariate analysis |  |  |  |  |  |  |  |  |
| Median Age (range) |  |  |  |  |  |  |  |  |
| Pelvis primary, N (%) |  |  |  |  |  |  |  |  |
| Interventions |  |  |  |  |  |  |  |  |
| Time to local treatment (Sx and or RT), weeks |  |  |  |  |  |  |  |  |
| Median no. of chemotherapy cycles |  |  |  |  |  |  |  |  |
| Local Therapy (surgery), n (%) |  |  |  |  |  |  |  |  |
| Local Therapy (Radiation) |  |  |  |  |  |  |  |  |
| Local Therapy (Surgery + Radiation) |  |  |  |  |  |  |  |  |
| Outcomes |  |  |  |  |  |  |  |  |
| Survival |  |  |  |  |  |  |  |  |
|  | 3-year EFS (%) | P Value | 3-year OS (%) | P Value | 5-year EFS (%) | P Value | 5-year OS (%) | P Value |
| 6-15 weeks local therapy (all) |  |  |  |  |  |  |  |  |
| 16+ weeks (all) |  |  |  |  |  |  |  |  |
| 6-15 weeks (surgery alone) |  |  |  |  |  |  |  |  |
| 16+ weeks (surgery alone) |  |  |  |  |  |  |  |  |
| 6-15 weeks (RT alone) |  |  |  |  |  |  |  |  |
| 16+ weeks (RT alone) |  |  |  |  |  |  |  |  |
| 5-16 weeks (Sx+RT) |  |  |  |  |  |  |  |  |
| +16 weeks (Sx+RT) |  |  |  |  |  |  |  |  |
| Multivariate Analysis of Prognostic Features for EFS in Pediatric and Adult Patients |  |  |  |  |  |  |  |  |
| Paediatric patients (n=) |  |  |  |  |  |  |  |  |
| Adult patients (n=) |  |  |  |  |  |  |  |  |
| Pelvic Primary Tumour (n=) |  |  |  |  |  |  |  |  |
| Weeks to local treatment |  |  |  |  |  |  |  |  |

Supplementary Table 1. Quality assessment for the seven studies according to NHMRC Evidence Hierarchy and Newcastle-Ottawa Quality Assessment Form for Cohort Studies

| **Study** | **Title** | **NHMRC Level of Evidence** | **Risk of Bias (Newcastle Ottawa scale for cohort study)** | | | |
| --- | --- | --- | --- | --- | --- | --- |
|  |  |  | **Selection** | **Comparability** | **Outcome** | **Overall** |
| Gupta 2010 | Clinical outcome of children and adults with localized Ewing sarcoma | III-3 | 4 | 2 | 2 | Good Quality |
| Ali 2014 | Outcome of Ewing sarcoma in a multidisciplinary setting in Lebanon | III-3 | 4 | 2 | 2 | Good Quality |
| Nasaka 2016 | Impact of treatment protocol on outcome of localized Ewing’s sarcoma | III-3 | 4 | 1 | 2 | Good Quality |
| Lin 2018 | Timing of Local therapy affects survival in Ewing sarcoma | III-3 | 4 | 2 | 3 | Good Quality |
| Lin 2020 | Relationship between treatment cancer case volume for localized Ewing Sarcoma: The role of radiotherapy timing | III-3 | 4 | 0 | 3 | Poor Quality |
| Parambil 2020 | Outcomes with nondose-dense chemotherapy with Ewing sarcoma: A practical approach for the developing world | III-3 | 4 | 2 | 2 | Good Quality |
| Totadri 2020 | Challenges in the management of localized Ewing sarcoma in a developing country | III-3 | 4 | 0 | 3 | Poor Quality |
| Heesen 2023 | Association between local treatment modalities and event-free survival, overall survival, and local recurrence in patients with localised Ewing Sarcoma. Report from the Ewing 2008 trial | III-3 | 4 | 2 | 3 | Good Quality |

Supplementary Table 2. NHMRC Evidence Statement

| **1. Evidence base** | **C.** One or two Level III studies with a low risk of bias or Level I or II studies with a moderate risk of bias |
| --- | --- |
| **2. Consistency** | **B**. All studies consistent |
| **3. Clinical impact** | **B.** Moderate |
| **4. Generalisability** | **B.** Evidence directly generalisable to target population with some caveats |
| **5. Applicability** | **B.** Evidence applicable to Australian healthcare context with few caveats (absence of Australian data, but there is no reason to the overseas data are not applicable in Australia) |
